# Behavioral rhythm and EEG rhythm to determine timing deficits in attention deficit hyperactivity disorder symptoms

**DOI:** 10.1016/j.heliyon.2020.e04546

**Published:** 2020-07-29

**Authors:** Shoko Kinumaki, Eri Miyauchi, Masahiro Kawasaki

**Affiliations:** Department of Intelligent Interaction Technology, Graduate School of Systems and Information Engineering, University of Tsukuba, Japan

**Keywords:** ADHD, Timing deficit, EEG, Error-related negativity, Synchronization, Beta, Cognitive neuroscience, Mental disorder, Psychiatry, Cognitive psychology, Personality, Neuroscience

## Abstract

One characteristic of attention deficit hyperactivity disorder (ADHD) is a timing deficit, i.e. difficulty tapping a self-selected pace and keeping the pace. The timing disorder is reported to relate to the frontal brain area. However, optimal means for evaluating this timing deficit and the corresponding neural mechanisms that accompany ADHD symptoms have not been identified. To address the issue, we required participants to tap one key of a keyboard sequentially and to maintain arbitrary tempos of their tapping intervals. We assessed ADHD symptoms using the Adult ADHD Self-Report Scale (ASRS) and evaluated brain activity via electroencephalography (EEG). Behavioral results indicated that the high ASRS group displayed a large inter-tap-interval gap (defined as the distribution of the time difference between the current tapping interval and the last one). Moreover, EEG results indicated that the work-load related brain activity (i.e. frontal beta activity) was higher in the high ASRS group. These results suggest that our tasks and analyses are useful for the evaluation of ADHD symptoms, although it was preliminary due to the small sample size and the non-patient data.

## 1 Introduction

One characteristic of attention deficit hyperactivity disorder (ADHD) is a timing deficit [1], that is, difficulty in tapping with keeping a self-selected pace [2] as well as with synchronizing an external stimulus rhythm [3]. Due to such timing deficits, ADHD patients experience difficulty predicting timing during a sensory task [4], difficulty in tapping a button in synchronization with a metronome in a motor task [5], and difficulty predicting motion in a visual stimulus in a sensory-motor task [2].

Previously, the ability to synchronize with external timing has been evaluated by using the timing variability in tapping tasks in which participants are required to tap along with a metronome or to tap keeping with a pace [6]. ADHD patients display a high degree of variability in timing. In contrast, some studies indicate no difference in timing variability in ADHD patients compared to healthy controls [7]. Moreover, it is unclear whether the timing deficits vary by the hand they used. Therefore, the utility of timing variability for the evaluation of ADHD symptoms remains debatable. As an alternative, this study investigates the utility of the inter-tap-interval gap which is defined as the time difference between the current inter-tap-interval and the prior inter-tap-interval, when participants are instructed to keep the inter-tap-interval constant [8].

The neural mechanisms involved in timing disorder are believed to be based in frontal areas, the basal ganglia and the cerebellum [9, 10]. Previous research utilizing an electroencephalogram (EEG) indicate frontal error-related negativity (ERN) when the tapping rhythm deviates from the expected rhythm [11] and as such is suggested to be one neural index of timing deficits. Moreover, when autism spectrum disorder (ASD) patients experienced difficulty synchronizing with others, they displayed high theta amplitudes, which are related to cognitive demand [12]. In contrast, readiness potentials which are observed in the motor areas during motion [13], represent tapping accuracy and complexity [14]. Given these previous findings, we hypothesized that frontal and motor activity during the tapping tasks would be an index of the timing-related ADHD symptoms.

Here, we attempted to identify the behavioral and neural indices of the timing disorder related to ADHD symptoms. To address this issue, we examined the relationship between ADHD symptoms and inter-tap interval and EEG activities during the tapping tasks which entail a request to tap at arbitrary paces constantly and continuously. The EEG data were analyzed using event-related potentials (ERP) and time-frequency analyses. Moreover, to clarify the effects of the daily fluctuations and dominant hands on the timing deficits, we conducted the tasks using both right and left hands over three days and compared behavioral performance and EEG activities between the hands.

## 2 Methods

### 2.1 Participants

Fifteen right-handed participants (7 women and 8 men; 21.1 ± 2.4 years) with normal or corrected-to-normal vision and normal motor function completed all experiments (i.e., three days). All participants provided written informed consent prior to participation. The study was approved by the Faculty of Engineering, Information and Systems, Research Ethics Committee of the University of Tsukuba (in accordance with the Declaration of Helsinki). Data obtained from 2 participants were excluded from the analyses because their EEG data included background noise (i.e., standard deviation of EEG amplitudes were over 100 μV).

### 2.2 Assessment

The ADHD symptoms were evaluated using the ASRS Symptom Checklist, version 1.1 [15]. The ASRS consists of 18 questionnaires: nine probe inattention and nine hyperactivity/impulsivity [16, 17]. The ASRS cut off points and coefficient alpha for the total scores have been reported as 36 and 0.78, respectively [18]. We defined the high ASRS group (over 36) and low ASRS groups (under 36).

### 2.3 Tapping task

Throughout the task, participants were required to sit in a chair, keep their eyes closed, and keep their head position within a chin rest. The task required them to press a key on a keyboard (i.e., tap) with their right or left index finger 200 times sequentially maintaining a constant time interval between the current tap and the last tap. The time intervals were arbitrarily assigned. The timings were feedbacked with a beep sound through their earphones.

Each participant completed the 2 conditions (right hand and left hand conditions). The order of conditions was counterbalanced across participants. Each participant completed 3 separate sessions (one session/day).

### 2.4 Behavioral analysis

Behavioral performance was evaluated based on time intervals between tapping. We identified as outliers and hence removed the trials for which inter-tap intervals were above 3 times the standard deviation from that individual's average tapping intervals.

We calculated the mean, standard deviation, and variable coefficient of the tapping intervals. The variable coefficients were defined as the mean value divided by the standard deviation [6].

To analyze the inter-tap-interval gaps, we estimated the distribution of the time difference between the current tapping interval and the last one. The inter-tap-interval gap was defined as the appearance ratio of the time bin (e.g., the ± 1-ms inter-tap interval was calculated by the probability that the gap was from -1 ms to 1 ms).

In both analyses, we conducted Pearsons’ correlation analyses between these data and ASRS scores and t-tests between high-ASRS and low-ASRS groups (no correction for multiple comparisons).

### 2.5 EEG recording

EEG data were recorded using 28 scalp electrodes (Ag/AgCl), in accordance with the extended version of the international 10–20 system in an electronic- and sound-shield room. EEGs were amplified using SynAmp2 (Neuroscan, El Paso, TX, USA) for 3 participants and using BrainAmp DC (Brain Product, Germany) for 12 participants (sampling rates: 1000 Hz). The reference electrodes were placed over the left and right mastoid. The impedance was below 14.6 kΩ.

### 2.6 EEG analysis

EEG data were analyzed using MATLAB software (Mathworks, Natick, MA, USA). EEG data for the reproduction period were segmented into 450-msec epochs from 150-msec before and 300-msec after the onset of each tap. EEG data were filtered with 0.5 Hz high pass and 40 Hz low pass filters and a 50 Hz notch filter.

In the ERP analyses, the peak value of the ERN component was calculated as the mean value of the EEG data at the frontal electrode (Fz). In contrast, the peak value of the motor readiness component was calculated as the mean value of the EEG data at the central electrode (Cz). We subtracted the baseline amplitudes which was defined as the averaged amplitudes from -100-msec to 0-msec from the onset of each tap. The ERP latency was calculated as the time point of the max value of the participants-averaged amplitudes for each ERP component. Next, we calculated each participant's amplitude at the latency and averaged them.

In the time-frequency analyses, the amplitudes were calculated with wavelet transforms using the Morlet wavelet function *w* (*t*, *f*_0_) [19]. Morlet wavelets *w* (*t*, *f*_0_) have a Gaussian shape around their central frequency *f*_0_ in both the time domain (SD σ_t_) and frequency domain (SD σ_f_).

$$\begin{array}{l} w\left(t,f\right)={\left(\sigma_{t}\sqrt{\pi }\right)}^{-\frac{1}{2}}\;\exp\left(-t^{2}/2\sigma_{t}^{2}\right)\exp\left(i2\pi ft\right)\text{,}\\ E\left(t,f\right)={\left|w\left(t,f\right)⊗s\left(t\right)\right|}^{2} \end{array}$$

where σ_f_ = 1⁄(2πσ_t_).

We used a wavelet that was characterized by a constant ratio (f⁄σ_f_ = 2), with *f* ranging from 1–30 Hz (0.5-Hz steps). The time-frequency amplitude *E* (*t*, *f*_0_) for each time point of each trial was the square norm of the convolution of a complex wavelet *w* (*t*,*f*_0_) with the original EEG signals *s*(*t*).

## 3 Results

### 3.1 ASRS

The high-ASRS and low-ASRS groups included 6 and 9 participants, respectively. The average total scores were 31.8 ± 11.1. The average inattention and hyperactivity/impulsivity scores were 18.9 ± 6.6 and 12.9 ± 5.1, respectively. There was significant correlation between the sub-scores (*r* = 0.79; *p* < 0.01).

### 3.2 Behavioral performance

There were no significant differences in timing variability for the average standard deviation, and variable coefficient between high-ASRS and low-ASRS groups, irrespective of the right/left hands conditions (*p* > 0.05). Moreover, there were no significant correlations between the timing variability and ASRS scores (*p* > 0.05).

**Figure 1:**
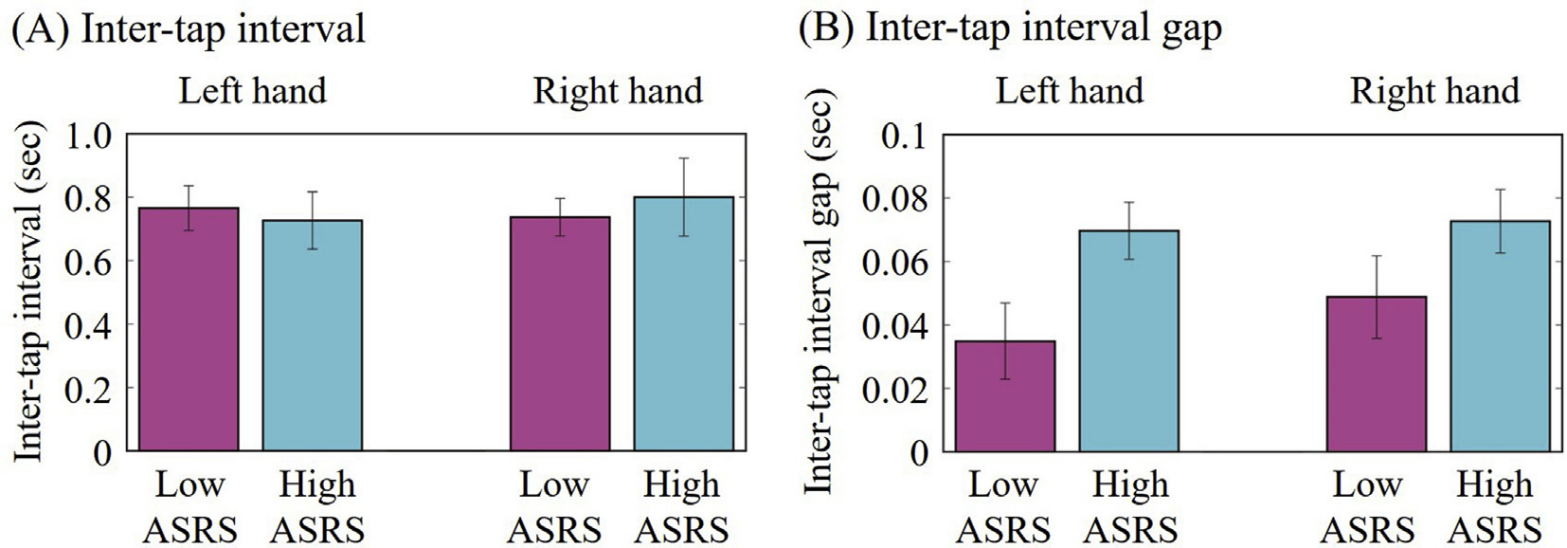
The participants-averaged inter-tap-interval (A) and the participants-averaged inter-tap-interval gap (i.e., the time difference between the current tapping interval and the last one) under left and right hand conditions for the low-ASRS (left) and high-ASRS (right) groups.

**Figure 2:**
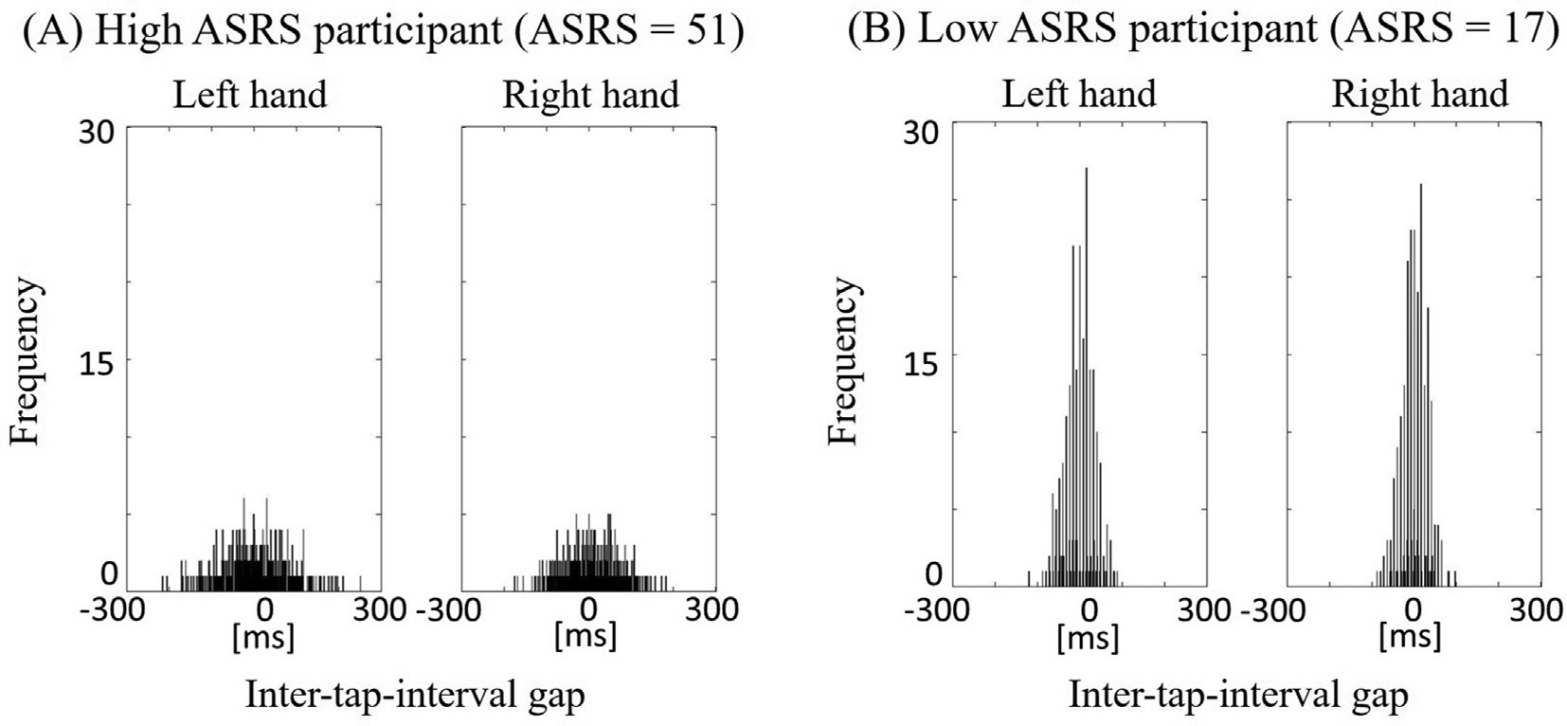
The histograms of the occurrence frequencies (i.e., number of tapping) for the time bins of the inter-tap-interval gap for the representative high-ASRS (A) and low-ASRS (B) individuals.

The averaged inter-tap intervals are shown in Figure 1A. In the inter-tap interval lag analyses, the histograms for the representative high-ASRS and low-ASRS individuals are shown in Figure 2. We calculated the probability of the ± 1-ms (i.e., time-bin) inter-tap interval gaps. The averaged inter-tap interval gaps are shown in Figure 1B. There were significant differences for the probabilities between high-ASRS and low-ASRS groups for the left hand condition (*t* = -2.40; *p* < 0.05) but not the right hand condition (*t* = -1.24; *p* > 0.05). Moreover, there were significant correlations between the inter-tap interval gaps and ASRS scores for the left hand condition (*r* = -0.66; *p* < 0.05) but not the right hand condition (*r* = -0.45; *p* > 0.05).

To investigate the time-bin that displayed significances we conducted t-tests and the correlation analyses under the left-hand condition by manipulating the probabilities of the inter-tap interval gaps. There were significant correlations between the inter-tap interval gaps and ASRS scores (±2-msec, *r* = -0.54, *p* < 0.05; ± 3-msec, *r* = -0.51, *p* > 0.05) and significant differences between groups (±2-msec, *t* = -1.83, *p* < 0.05; ± 3-msec, *t* = -1.75, *p* > 0.05) under the ± 2-msec test.

### 3.3 EEG results

**Figure 3:**
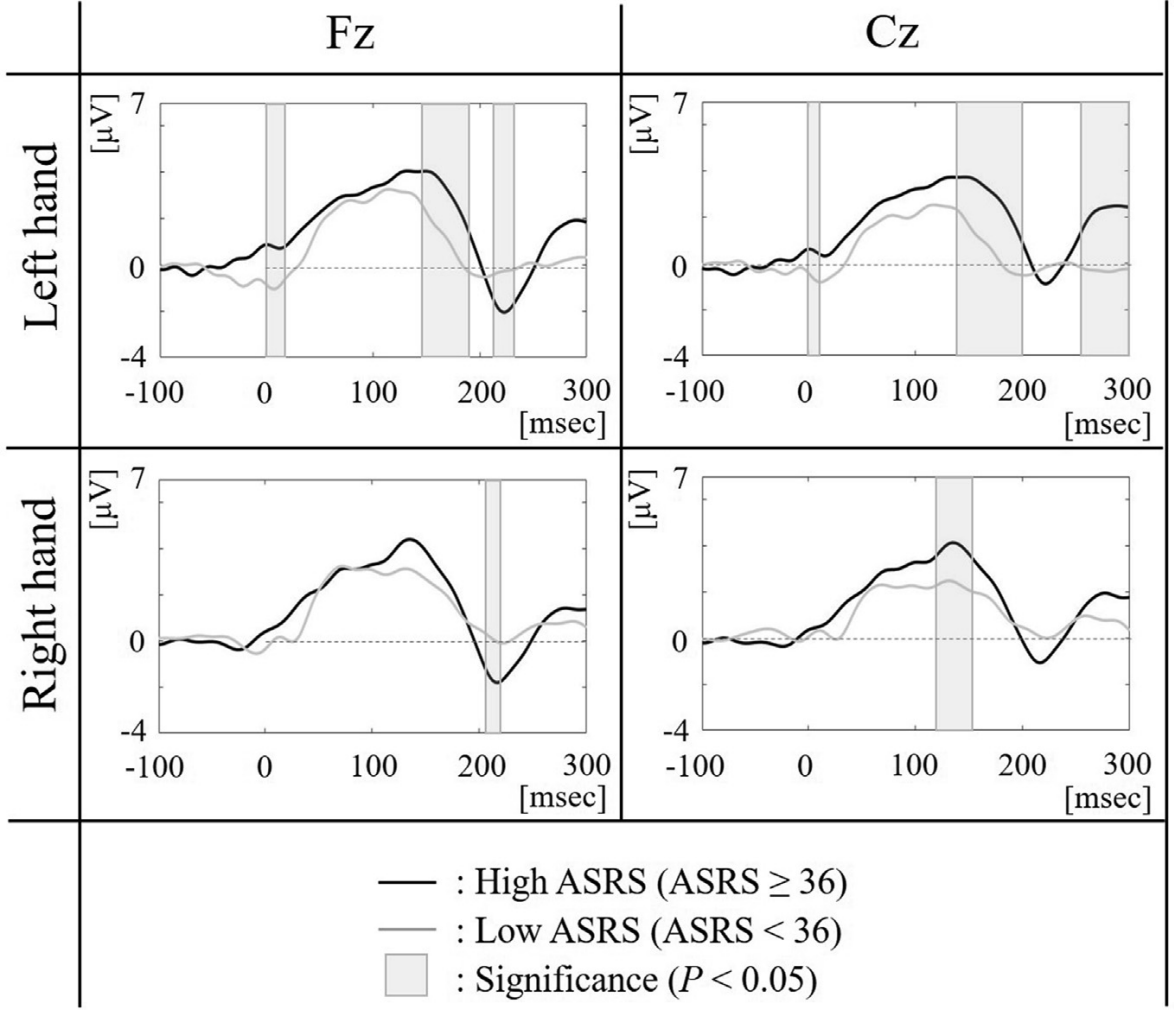
The Fz and Cz ERPs of the high-ASRS (thick line) and low-ASRS (thin line) groups around the tapping for the right and left hand conditions. The 0-msec means the onset of the tapping. The gray highlighted time-periods indicate the significant differences between them (*p* < 0.05).

We focused on the Fz and Cz electrodes as the frontal and motor areas, respectively. The ERPs of the high-ASRS and low-ASRS groups for the right and left hands conditions are shown in Figure 3. The gray highlighted time-periods indicated significant differences (*p* < 0.05; no correction for multiple comparisons). The latency of the first negative peak activity of Fz electrodes was evident approximately 220 ms after tapping onset (Table 1a). The minimum amplitude for each participant was computed in the time-period 100–300 ms from the onset of tapping, as the negative peak Fz amplitude. The negative peak Fz amplitudes of the high-ASRS group were significantly lower than those of the low-ASRS group for both the right hand (*t* = -1.83, *p* < 0.05) and left hand conditions (*t* = -1.85, *p* < 0.05).

**Table 1:** The latency and the group-averaged peak amplitude of (a) the first negative peak activity of Fz electrodes and (b) the first positive peak activity of Cz electrodes.

condition	group	latency	peak amplitude
(a) The first negative peak activity of Fz electrodes			

Left hand	High ASRS	222 ms	-2.05 ± 0.78
	Low ASRS	213 ms	-0.52 ± 0.46

Right hand	High ASRS	218 ms	-1.81 ± 0.74
	Low ASRS	223 ms	-0.10 ± 0.54

(b) The first positive peak activity of Cz electrodes			

Left hand	High ASRS	147 ms	3.74 ± 0.60
	Low ASRS	127 ms	2.53 ± 0.55

Right hand	High ASRS	135 ms	4.14 ± 0.58
	Low ASRS	131 ms	2.49 ± 0.56

In contrast, the latency of the first positive peak activity of Cz electrodes was evident approximately 150 ms after tapping onset (Table 1b). The maximum amplitude for each participant was computed in the time-period 100–300 ms from the onset of tapping, as the positive peak Cz amplitude. The positive peak Cz amplitudes of the high-ASRS group were significantly higher than those of the low-ASRS group for both the right hand (*t* = 1.88, *p* < 0.05) and left hand conditions (*t* = 1.80, *p* < 0.05).

**Figure 4:**
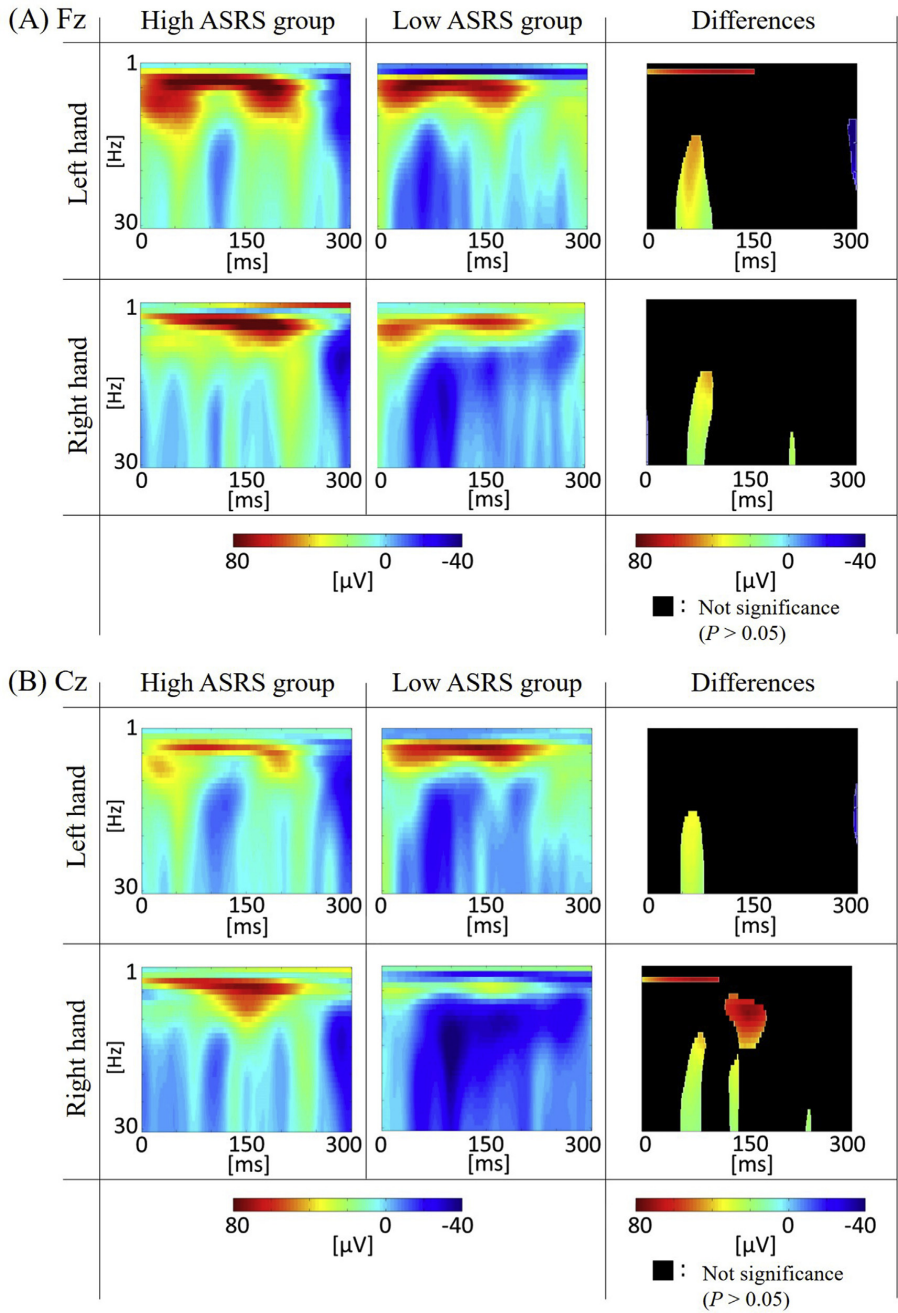
The averaged time-frequency amplitudes on the Fz (A) and Cz (B) electrodes of the high-ASRS (left panel) and low-ASRS (central panel) groups around the tapping for the right and left hands conditions. The right panel shows the different amplitudes of the high-ASRS group minus the low-ASRS group in only the time-frequency epochs showing significant differences between them (*p* < 0.05). The black areas indicate the time-frequency epochs showing no significance. The participants tap at 0-msec time point.

The averaged time-frequency amplitudes of the high-ASRS and low-ASRS groups for the right and left hands conditions displayed significant differences between the groups (*p* < 0.05; no correction for multiple comparisons) (Figure 4 right panel). In the left-hand condition, the frontal and motor beta-band (15–30 Hz) amplitudes for the high-ASRS group were significantly higher than those for the low-ASRS group around 80 ms after tapping onset in both the right and left hand conditions. In addition to the beta amplitudes, only the motor areas showed the higher theta amplitudes for the high-ASRS group than for the low-ASRS group around 80 ms after tapping onset under only the right hand condition.

## 4 Discussion

### 4.1 Behavioral performance

Our findings demonstrate the usefulness of inter-tap intervals rather than the timing variability during the tapping task for the assessment of ADHD symptoms. Inter-tapping intervals indicate significant differences between the high-ASRS and low-ASRS groups and a significant correlation with ASRS scores. These results are consistent with previous findings that the timing deficits of the ADHD symptoms reflect difficulty in keeping one's own individual pace [2] as well as in synchronizing time intervals with external stimulus [3].

The inter-tapping intervals could be calculated by using only 2 intervals; interval between the current and previous one taps and interval between the previous one and previous two taps. In contrast, the average, standard deviation, and timing variability are calculated by integration, specifically requiring a sufficient number of the taps. Furthermore, these values used in previous research [2, 6] failed to demonstrate a significant relationship with the ASRS symptoms in this study. Taken together, previous evidence and our current findings support the inter-tap interval as useful for the estimation of the stability of behaviors associated with ADHD symptoms.

Moreover, the significant differences we observed were specific to the left hand which was the non-dominant hand for our participants. The differences between the right and left hands motions were reported to be represented by the motion-related brain potentials [11]. ADHD patients demonstrated difficulty using their nondominant hand in comparison with the healthy participants [20]. Therefore, it is important to assess individual handedness when evaluating ADHD symptoms.

### 4.2 EEG results

Our findings demonstrate that frontal ERN amplitudes represent the timing deficits of the ADHD symptoms, since the first negative peak activities in the frontal electrode after the tapping (i.e., ERNs) for the high-ASRS group were significantly lower than that for the low-ASRS group. This result for ERP components is consistent with other studies showing that the ERN is generated from the midline frontal cortex [21]. The frontal ERN is thought to be associated with error monitoring [22] and error adaptation [23, 24]. Moreover, previous studies have also reported the relationship between ERN and ADHD symptoms [25, 26]. Although those studies employed sensory-response tasks, the current study also found the frontal ERN for the self-pace-constant tapping task. In this study, the ERN events might be considered as not the error but the large inter-tap interval gaps. Therefore, the brain activity of the high ASRS group could be an unconscious response to the tapping error, in comparison with the low ASRS group.

The central positive activity at 150 ms from the tapping onset as the motor readiness potential may reflect the task difficulties that the high ASRS group experienced while maintaining the pace of tapping. The ERP amplitudes were significantly higher for the high ASRS than for the low ASRS group under both the right and left hand conditions. These results are consistent with previous research that indicates the ERP components are reportedly involved in motor accuracy and complexity [14].

In our time-frequency analyses, we observed that the frontal beta amplitudes indicate the high task demands of the ASRS group during tapping tasks. At 80 ms from the tapping onset, the frontal beta amplitudes were significantly higher for the high ASRS than for the low ASRS groups under both the right and left hand conditions. Previous research indicates that the frontal beta amplitudes are likely involved in task demand [27] and the level of the top-down control of behavior [28]. Moreover, the ADHD patients displayed the same increment of the frontal beta amplitudes in the mental and physical tasks [29].

In previous studies, the theta activity was related to the ADHD symptoms [29, 30, 31]. However, the ADHD-related theta amplitudes were found under the right hand condition but not under the left hand condition, which was related to the ADHD symptoms. The previous studies used different tasks than ours; the detection task [29], oddball task [30], and decision-making task [31]. Therefore, it is possible that the high-ASRS group was better able to maintain tapping with the right-hand but not with the left hand, leading to no difference in performance compared to the low-ASRS group. Furthermore, there are considerable EEG studies showing the increase theta/beta ratio (i.e., increase of theta power and decrease of beta power) in ADHD during the eye-open states (e.g., [25]). The increased theta power in the high-ASRS group in our study is consistent with such findings. However, the beta power of the high-ASRS group also increased, which was opposite to previous finding. It might be due to our study focusing on the simple motor ability in ADHD symptoms, unlike previous studies. Therefore, the beta activity might be different between our study and previous studies. To elucidate the findings of our EEG results, future research should simultaneously conduct not only the tapping task but also the high task demand task for the ADHD patients.

As a limitation, our participants had not received the medical diagnoses for ADHD. Furthermore, our statistics showed no significances in the multiple comparison analyses. Therefore, it is noted that our results were preliminary. In future study, a large number of participants should be included and the comparison between patients and controls should be conducted.

## 5 Conclusion

In conclusion, our results suggest that the inter-tap-interval gap and the work-load related EEG activity (i.e. frontal ERN and frontal beta activity) represent the behavioral index and the EEG index of the ADHD symptoms, respectively.

## Declarations

### Author contribution statement

Shoko Kinumaki, Eri Miyauchi, Masahiro Kawasaki: Conceived and designed the experiments; Performed the experiments; Analyzed and interpreted the data; Wrote the paper.

### Funding statement

This research did not receive any specific grant from funding agencies in the public, commercial, or not-for-profit sectors.

### Competing interest statement

The authors declare no conflict of interest.

### Additional information

No additional information is available for this paper.
